# Pneumatic Forceps

**Published:** 1878-08

**Authors:** 


Pneumatic Forceps.—At a recent meeting of the Boston Obstetrical Society, a forceps was shown—the invention of a Dr. Stillman, of Usquepaugh, R. I.—which consists of a cup, to be applied to the fetal head, and an exhaust-syringe; the whole, when in place and the air exhausted, being capable of supporting about seventy pounds.

A student at the Columbus Medical College last spring presented a thesis proposing a similar instrument, and defended it quite ingeniously. The danger, of course, consists in injuring the scalp and causing abscess. This occurred in one case in the practice of Dr. S., which, at the time the report was made, had been limited to three cases. It is said that Prof. Simpson, of Edinburgh, devised a similar instrument twenty years ago.—*Ohio Medical Recorder.*

